# Malnutrition in Eosinophilic Gastrointestinal Disorders

**DOI:** 10.3390/nu13010128

**Published:** 2020-12-31

**Authors:** Martina Votto, Maria De Filippo, Francesca Olivero, Alessandro Raffaele, Emanuele Cereda, Mara De Amici, Giorgia Testa, Gian Luigi Marseglia, Amelia Licari

**Affiliations:** 1 Pediatric Clinic, Department of Pediatrics, Fondazione IRCSS-Policlinico San Matteo, University of Pavia, 27100 Pavia, Italy; martinavotto@gmail.com (M.V.); maria_defilippo@hotmail.it (M.D.F.); francescaolivero31@gmail.com (F.O.); m.deamici@smatteo.pv.it (M.D.A.); giorgia_testa@hotmail.it (G.T.); gl.marseglia@smatteo.pv.it (G.L.M.); 2 Pediatric Surgery Unit, Department of Maternal and Child Health, Fondazione IRCCS Policlinico San Matteo, 27100 Pavia, Italy; a.raffaele@smatteo.pv.it; 3 Clinical Nutrition and Dietetics Unit, Fondazione IRCCS Policlinico San Matteo, 27100 Pavia, Italy; e.cereda@smatteo.pv.it; 4 Immuno-Allergology Laboratory of the Clinical Chemistry Unit, Fondazione IRCCS Policlinico San Matteo, 27100 Pavia, Italy

**Keywords:** children, adolescents, eosinophilic esophagitis, eosinophilic gastrointestinal disorders, growth, failure to thrive, malnutrition, undernutrition, obesity, vitamin

## Abstract

Primary eosinophilic gastrointestinal disorders (EGIDs) are emerging chronic/remittent inflammatory diseases of unknown etiology, which may involve any part of the gastrointestinal (GI) tract, in the absence of secondary causes of GI eosinophilia. Eosinophilic esophagitis is the prototype of eosinophilic gastrointestinal disorders and is clinically characterized by symptoms related to esophageal inflammation and dysfunction. A few studies have assessed the nutritional status of patients with eosinophilic gastrointestinal disorders, showing conflicting results. This review summarizes the current evidence on the nutritional status of patients with EGIDs, focusing on the pediatric point of view and also speculating potential etiological mechanisms.

## 1. Introduction

Primary eosinophilic gastrointestinal disorders (EGIDs) are emerging chronic/remittent inflammatory diseases of unknown etiology, which may involve any part of the gastrointestinal (GI) tract, leading to eosinophilic mucosal infiltration in the absence of secondary causes of intestinal eosinophilia [1,2,3]. While eosinophilic esophagitis (EoE) is a well-characterized disease with established guidelines [4,5], nonesophageal EGIDs, including eosinophilic gastritis, gastroenteritis, and colitis, remain a clinical enigma [1]. Although their pathogenic mechanisms are still unknown, EGIDs seems to be commonly associated with atopy and, to a lesser extent, autoimmunity [1,2]. EoE pathogenesis has been more extensively studied, and advances concerning the genetic and environmental contributors and cellular and molecular etiology have been achieved [6]. EGIDs seem to be multifactorial diseases resulting from genetic predisposition, environmental risk factors, and intestinal dysbiosis, leading to the activation of T-helper type 2 (Th2) inflammation and impaired epithelial barrier [1,7]. To date, no studies have extensively assessed malnutrition in patients with EGIDs.

In all its forms, malnutrition includes undernutrition, inadequate intake of vitamins and/or minerals, overweight, and obesity [8]. Undernutrition is a common complication of several chronic inflammatory GI diseases, mainly coeliac disease (CD) and Crohn’s disease, often associated with weight loss, failure to thrive, malabsorption, and vitamin deficiency. However, obesity and overweight are the main comorbidities of gastroesophageal reflux disease (GERD) and functional GI disorders, and are well-known risk factors of hepatic steatosis [9,10]. 

This review aims to summarize the current evidence on the nutritional status and malnutrition in patients with EGIDs, mainly focusing on the pediatric patients’ population and highlining the lack of nutritional management algorithms. 

**Figure 1:**
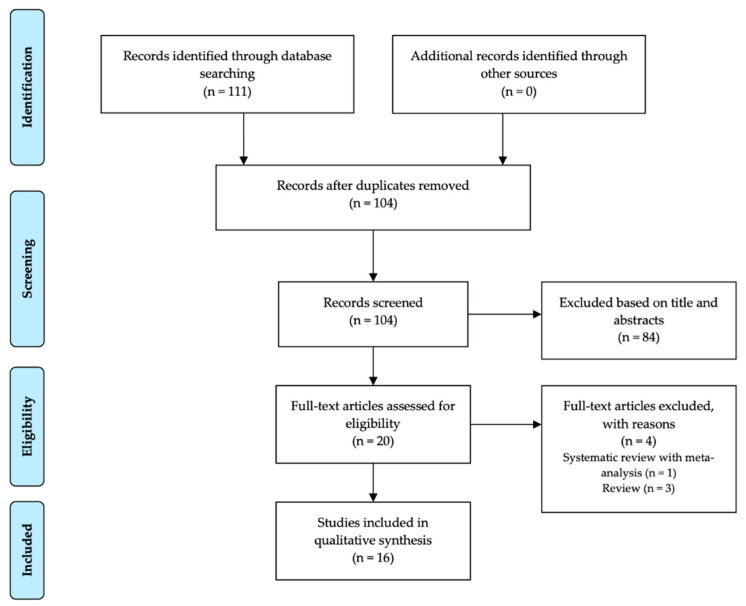
Process of literature screening.

A review of articles was performed via the online database PubMed (Table 1), following PRISMA guidelines [11]. The literature review was performed in December 2020, including all publication years. All studies that met the following criteria were included: (1) case reports, case series, and cross-sectional and cohort studies published in English in peer-reviewed journals; (2) participants were children and adult patients diagnosed with EGIDs. Potentially eligible publications were manually screened and reviewed, and nonrelevant publications were excluded (Figure 1). 

## 2. Obese and Overweight EGID Patients

Obesity is a global public health problem associated with many chronic diseases, including type 2 diabetes, arterial hypertension, cardiovascular diseases, and asthma [12]. Growing evidence supports the association between obesity and immune disorders, such as cancer, autoimmunity, and atopy [13]. Some studies have suggested that pediatric obesity epidemy and obesity-related inflammation might at least in part be responsible for the significantly raised prevalence of allergic diseases [13]. The relationship between asthma and obesity in children is widely demonstrated, and several observational studies have reported that obese children are more frequently affected by a severe phenotype of asthma, refractory to conventional therapies [14,15,16,17]. Additionally, data from the National Health and Nutrition Examination Study III (NHANES III) have described a positive association between body mass index (BMI) and atopy rates [17]. However, a real link between obesity and other allergic disorders, such as allergic rhinitis, atopic dermatitis, as well as EGIDs, has not yet been extensively established [18]. A few studies have assessed the role of body weight and BMI in children and adolescents with EoE, and no articles were published on EGIDs distal to the esophagus (Table 2). There is evidence that most adults with EoE mainly have a good nutritional status and expected BMI values [19,20,21,22,23,24,25,26,27]. Despite feeding or swallowing issues, EoE children did not generally report nutritional deficiency or impaired growth [23]. Rezende et al. found that 82.8% of the enrolled EoE children had a good nutritional state, 11.4% were overweight, whereas 5.7% were underweight [27]. Moreover, Jensen et al., 2019 reported that EoE children might present a slight impairment of height at diagnosis and achieve their expected growth, regardless of treatment modality [21]. Finally, children with GERD and EoE had a weight-for-length (WFL) Z score at the 18th–13th percentiles; thus, they did not meet the criteria for failure to thrive (FTT) [24].

To date, no research has investigated the possible pathogenetic role of obesity in EGID development. Putative explanations could probably be found in environmental and genetic risk factors and EGID-related comorbidities. The overall prevalence of EGIDs seems to higher in developed Western countries, where childhood obesity and atopic diseases were significantly increased through time [7,28]. Indeed, obesity and the Western lifestyle, mainly characterized by high calorie/fat consumption and reduced physical activity, might be directly related to the increased risk of developing allergic diseases, such as EGIDs [13]. In a study in mice, Silva et al. demonstrated that obesity aggravated the immune histopathological characteristics of the EoE experimental model, reducing the regulatory cytokines profile (low expression of forkhead box P3, FOXP3, and interleukin 10, IL-10), increasing the inflammatory mediators (IL-5 and thymic stromal lymphopoietin, TSLP), and promoting tissue remodeling [29]. These fascinating data might provide new insights about obesity as a possible EoE risk factor that might impair esophageal inflammation and symptoms. 

Another possible pathogenetic mechanism might be the relationship between EoE and GERD. Diagnosis of GERD has also increased, especially in developed countries [7]. In half of the infants with refractory vomiting and regurgitation, GERD was also expressed in the underlying cow’s milk allergy, and improved with a hydrolyzed formula [30]. Several studies reported that GERD might play a possible pathogenetic role in esophageal eosinophilia, more relevant in PPI-responsive patients [31]. Indeed, EoE and GERD are not mutually exclusive and might coexist [4]. Although there are no exact data, four mechanisms have been proposed to explain this association: (1) GERD only causes esophageal eosinophilia; (2) GERD and EoE coexist but are independent phenomena; (3) EoE induces GERD; (4) GERD contributes to or induces EoE [7,31]. Acid reflux alters the esophageal epithelial barrier, leading to high intestinal permeability, with a subsequent passage of food allergens and release of inflammatory and eosinophil chemoattractant molecules might trigger EoE in susceptible subjects [32]. 

On the other hand, the esophageal eosinophilic inflammation is also associated with the production of different proinflammatory cytokines that might impair peristalsis and the esophageal acid clearance [7,33]. The subepithelial fibrosis, a delayed complication of EoE, might also promote esophageal dysmotility and GERD-related symptoms [31]. It is well described that being overweight and obese contribute to the development and worsening of GERD frequency and symptoms [34,35]. Obesity is notoriously involved in the pathogenesis of GERD [23]. Visceral fat might mechanically induce reflux events, increasing the intra-abdominal pressure [36]. Additionally, abdominal fat is metabolically active, activating macrophages, increasing and releasing proinflammatory cytokines and adipokines such as leptin [23,36]. 

Genes, obesity, and atopic diseases are linked. This association is well described in asthma patients, whereas no studies have been reported on EGID subjects. The β2-adrenergic (ADRB2) and glucocorticoid (NR3C1) receptor genes have been involved in the development of asthma and obesity [13]. Similarly, polymorphisms of the fractalkine receptor gene (CX3CR1) have been associated with asthma, atopy, and obesity [16]. However, no studies have described a genetic correlation between obesity/overweight and EGIDs. 

Finally, EoE is characterized by chronic inflammation, specifically affecting the esophagus and generally sparing other GI tracts. This feature could clarify why EoE is not related to intestinal malabsorption and does not affect the bodyweight of adult patients. 

The relationship between EGIDs, overweight, and obesity is still speculative, and further studies are required to confirm these clinical findings. 

## 3. Undernutrition and Failure to Thrive in EGIDs Patients

Although poorly investigated, EGIDs may also be complicated by undernutrition and FTT for pathogenetic mechanisms similar to those reported in inflammatory bowel disease (IBD) patients [37]. FTT is one of the most commonly described clinical complications in children with EoE [3,38], although the exact prevalence has never been documented. Retrospective studies have reported that the prevalence of FTT ranges from 10.5% to 24% of EoE patients with different age-related rates (Table 3) [39,40,41,42,43,44]. In a large retrospective study, Spergel et al. demonstrated that FTT mainly characterized young children (2.8 ± 3.2 years) [44]. Moreover, Alhmoud et al. reported FTT and weight loss only in children with EoGE, and 15% of these had severe mucosal involvement leading to malabsorption [41].

Several factors may negatively impact the nutritional status of EGIDs patients (Table 4), mostly children. Firstly, children with EoE more likely present feeding disorders, recurrent vomiting, or regurgitation due to the esophageal inflammation and dysfunction, which can severely impair the adequate intake of foods and nutrients [2,3]. EGIDs are emerging GI disorders, therefore the diagnostic delay was often reported in adolescents and adults, who can consequently develop esophageal strictures due to the chronic inflammation and fibrous tissue deposition, prolonging clinical symptoms and patient feeding discomfort [45].

Secondly, low compliance to treatment is one of the main reasons for therapeutic failure and persistent active EoE, especially in adolescents and adults [46]. Chronic GI symptoms and impaired oral food intake, due to the sustained esophageal inflammation and continued low-grade antigen exposure, through limited dietary compliance are other possible explanations for undernutrition. 

Thirdly, children, adolescents, and adults with previous food impaction episodes may have a high risk of developing anxiety and eating disorders, such as nervous anorexia and food avoidance, leading to an inadequate nutrient intake [46,47]. In a case-control study, Wu et al. found that most children with EGIDs had feeding behavioral problems compared to healthy controls [48]. Another study showed that 16.5% of EGID children had feeding issues, such as food refusal, low volume, and variety of intake, grazing, and spitting food out [49]. Moreover, 21% of these children were also complicated by FTT, suggesting that feeding issues may impair the regular childhood oral intake contributing to undernutrition and growth failure [49]. 

Additionally, a retrospective multicentric U.S. study of Consortium of Eosinophilic Gastrointestinal Disease Researchers (CEGIR) reported that 41% of children and adolescents with nonesophageal EGIDs might have a multisite GI inflammation [50]. This finding suggests that the persistent GI inflammation and subsequent abnormal intestinal permeability may be possible reasons for nutrients loss and higher caloric and protein requirements in patients with EGIDs distal to the esophagus [24].

Moreover, the association between EoE and other allergic conditions is well established and might be a potential further reason for FTT and undernutrition in EGIDs children. Children with EGIDs have an excessive prevalence of atopic dermatitis, IgE-mediated food allergy, asthma, and allergic rhinitis, potentially affecting the expected growth [51]. Moreover, several reports have suggested that EGIDs may also be frequently associated with chronic non-allergic comorbidities that might compromise adequate child growth, feeding behavior, and quality of life [46]. In a cross-sectional study, Capuccilli et al. demonstrated that children with EoE also had higher rates of coexisting non-atopic diseases, including IBD (0.7%) and CD (5.6%), as well as a higher prevalence of autism spectrum disorders (ASDs) (7.5%), type 1 diabetes mellitus (1.2%) and cystic fibrosis (0.9%) [52]. 

Finally, an important unanswered question is whether therapies can influence FTT. Paquet et al. have reported that EoE-related FTT resolved in 62% of affected children, suggesting that medical interventions might be helpful not only for disease-remission but also for clinical complications [42]. However, these results cannot be generalized because this study was retrospective and based on a small number of patients (15 patients with EoE + FTT). On the other hand, it was widely described that impaired growth and inadequate intake of macro- and micronutrients are possible complications of restrictive food elimination diets, which are pivotal therapeutical approaches of several pediatric illnesses, including EGIDs [1]. Several clinical factors might induce protein–calorie malnutrition and impaired food intake with weight loss, FTT, and delayed puberty. These findings underly the importance of assessing potential risk factors that may bring dietary limitations and normal growth of children with EGIDs.

## 4. Vitamin D Deficiency in EGIDs

Low serum vitamin D levels have been proposed to explain the increased prevalence of atopic and autoimmune diseases in Western countries [53]. Several efforts have focused on the role of vitamin D in the contribution of chronic dysregulated inflammation and its modulation [53]. Prevalence of EoE is higher in Western countries and cold climate zones, suggesting a possible association with low serum vitamin D levels [7]. Increasingly, significant evidence has shown a consistent link between vitamin D deficiency—due to the quality of diet, lack of exposure to sunlight—and the risk of atopy, as already described for asthma, allergic rhinitis, food allergy, and atopic dermatitis [7].

A systematic review has reported that low vitamin D prevalence varied widely in enrolled studies (0–52%) and did not improve with therapy [24,54] (Table 5). Low levels of vitamin D were described in 42% of adults and 50% of children with EoE, prevailing in patients with symptoms of food impaction [54,55]. In a case-control study of 69 children, Waterhouse et al. reported that patients with EoE and GERD had low vitamin D levels compared to normal controls, but without a significant difference [56]. To date, no study assessed other vitamins in EGIDs and serum vitamin D in patients with EGIDs beyond the esophagus. 

Although there is emerging evidence of vitamin D in the development of the immune system and pathogenesis of allergic diseases, such as asthma, atopic dermatitis, and food allergy, no studies have evaluated its possible role in EGIDs development and remission [53]. Furthermore, based on the design of available studies (cross-sectional data analysis) no cause–effect relationship can be inferred. It is reasonable to argue that toddlers and young children with EoE could present with feeding difficulty and refusal, with subsequent nutrient deficiencies, thus malnutrition. Besides, food elimination diets, mostly milk-free diets, could increase the risk of vitamin D deficiency in EoE patients, as reported in children with cow’s milk allergy [57,58]. 

## 5. Management of EGIDs Patients: From Traditional Tools and Treatments to Future Insights

Diagnoses of EGIDs are not always straightforward and require chronic GI symptoms, coupled with suggestive endoscopic findings, prevalent eosinophilic inflammation (≥15 eosinophils/high-power field (HPF) for EoE) in biopsy specimens, and the exclusion of other causes of GI eosinophilia [1,4,5]. Symptoms of EGID are generally heterogeneous and often overlap with other conditions and may occur concomitantly. In EoE, the eosinophilic inflammation leads to progressive esophageal dysfunction, mainly characterized by feeding refusal and vomiting in children, and dysphagia, heartburn, and food bolus impaction in adolescents and adult patients [3]. Patients do not always appear to have feeding or eating disorders; only 24% of younger patients showed a failure to thrive. As reported in this review, most patients were normal weight or even obese. A meticulous evaluation of the patient’s symptoms should be recommended, and the clinician should ask the right questions to detect suspicious eating habits (Table 6) [59].

Although several research efforts have produced fascinating progress in the diagnosis and management of EGIDs, especially EoE, the only currently available tool to confirm the clinical suspicion is GI endoscopy with a biopsy [4,5]. Nevertheless, surrogate measures for EoE activity and response to therapy, such as the esophageal String test, transnasal esophagoscopy, and Cytosponge, have emerged as effective, less invasive tools for obtaining esophageal tissue samples [60,61].

Since EoE was initially identified in the mid-1990s, multiple EoE treatment strategies have been developed. Dietary treatment represented the first-line therapeutical approach for EGIDs [1,4,5]. Elemental (exclusive amino acid-based formulas) and six-food (milk, wheat, egg, soy, fish and shellfish, nuts) elimination diet (SFED) are the two main nutritional methods for EGID management with high rates of remission [1,4,5]. Trials have reported that a significant proportion of EoE patients achieved histologic remission on less restrictive (two/four food elimination) diets. Thus, personalized dietary strategies might offer the greatest success, improving the nutritional status and quality of life of affected subjects [60]. Successful targeted removal of specific foods based on allergy tests have been reported as case reports. However, targeted food removal might not be effective and is not recommended, because response to therapy did not seem to correspond to food allergies identified by skin prick testing or measuring serum food-specific IgE concentrations [62].

Swallowed steroids are alternative EGID treatments to diet-based interventions. The two most common approaches include swallowed fluticasone and viscous budesonide [4,5]. Comparisons between elimination diets and swallowed steroids are difficult, due to the heterogeneity of available studies. Meta-regression analyses showed that both therapeutical approaches are generally equivalent at inducing histologic remission in EGIDs patients [63]. 

Unfortunately, a significant population of patients with EGIDs has persistent active disease. Therefore, several ongoing efforts identify promising biological therapies beyond diet or steroid strategies [60,64]. Future efforts should be targeted to particular EGID endotypes using traditional and biologic therapies to achieve a new and high disease control degree.

### How to Manage Malnutrition in Children with EGIDs?

**Figure 2:**
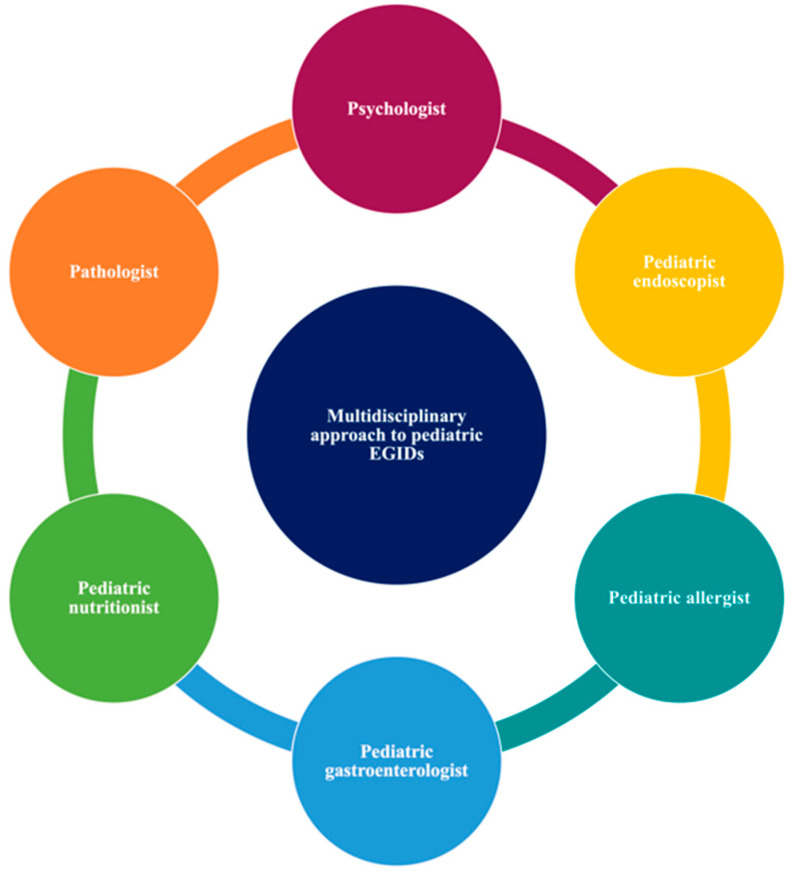
The multidisciplinary approach of children and adolescents with eosinophilic gastrointestinal disorders.

**Figure 3:**
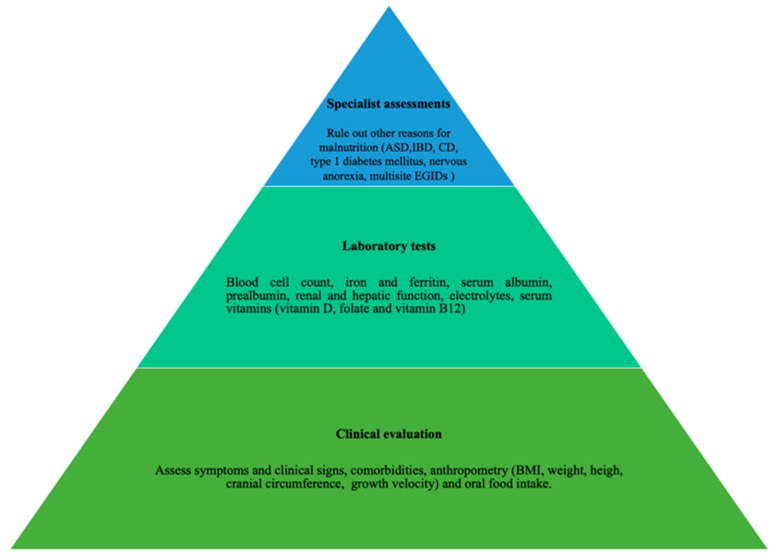
Nutritional status assessment of children and adolescents with eosinophilic gastrointestinal disorders.

This study suggests that a multidisciplinary approach (allergist, gastroenterologist, nutritionist, psychologist) is a key winner of EGIDs management (Figure 2), especially in children with allergic and non-allergic phenotypes. Moreover, the nutritional status assessment may help recognize patients with an inadequate nutrient intake, especially if they require restrictive food elimination diets (Figure 3). 

This review summarized evidence on pediatric EGIDs malnutrition and underly conflicting findings. While some studies have reported normal or high BMI, especially in adults with coexisting GERD, FTT might mostly afflict young children. As reported for allergic diseases, EGIDs may also show vitamin D deficiency. However, no study has assessed how intestinal inflammation or EGIDs therapies may impact serum vitamin D and bone metabolism. Despite an inadequate investigation, EGID malnutrition is a relevant clinical field that requires further efforts to strengthen the efficacy of therapies and improve the patients’ quality of life.

## Figures and Tables

**Table 1:** Search strategy.

PubMed: “Eosinophilic gastrointestinal disorders” AND “malnutrition.” Publication date: all years.
PubMed: “Eosinophilic gastrointestinal disorders” AND “obesity.” Publication date: all years.
PubMed: “Eosinophilic gastrointestinal disorders” AND “vitamin.” Publication date: all years.

**Table 2:** Studies reporting a normal or high BMI of children and adult patients with EoE. No study has been published on non-esophageal eosinophilic gastrointestinal disorders (EGIDs).

Author, Year	Country	Study Design	Sample Size	Population	Outcomes
Zdanowicz et al., 2020 [19]	Poland	Single-center retrospective study	36 EoE patients	Children	No difference was observed in the prevalence of failure to thrive between children with EoE and controls (30.6% vs. 19.14%).
Alexander et al., 2020 [20]	U.S.A.	Retrospective cohort study	223 EoE patients	Adults	PPI non-responding EoE patients were younger (*p* = 0.001), had a lower BMI (27.3 vs. 28.6 kg/m^2^, *p* = 0.04), and higher peripheral eosinophil count (*p* = 0.006) than responders, suggesting that these variables might be risk factors for PPI non-response in EoE.
Jensen et al., 2019 [21]	U.S.A.	Retrospective multicenter study	409 EoE patients	Children (<18 years)	Children with EoE had a slight impairment of height at diagnosis; thus, they were not malnourished. Additionally, they generally maintained their expected growth regardless of treatment modality. Subtle changes were noted for patients treated with elemental diets in combination with other therapeutical approaches.
Kovačić et al., 2019 [22]	Croatia	Cross-sectional study	32 EoE patients	Children (<18 years)	Most of the enrolled patients were well-nourished, and a normal BMI Z score was found in 75% of the patients. There was no difference in BMI Z score between baseline and 12 months follow-up (median −0.3 vs. −0.3 SD, *p* = 0.862).
Tanaka et al., 2019 [23]	Japan	Cross-sectional study	27 EoE patients	Adults	Subjects with EoE had higher BMI values than those without EoE (23.4 kg/m^2^ vs. 22.3 kg/m^2^, *p* = 0.005). Additionally, they had a higher proportion of bronchial asthma and hiatal hernia compared to controls (25.9% vs. 5.2%; *p* < 0.00129.6% vs. 14.7%; *p* = 0.049).
Mehta et al., 2018 [24]	U.S.A.	Prospective study	91 patients (GERD = 38, EoE = 53)	Children (0–7 years)	Children with GERD and EoE had greater eating issues than healthy controls and did not report nutritional deficiency or impaired growth. Additionally, children with GERD and EoE had a WFL Z score at the 18th and 13th percentiles; thus, they did not meet FTT criteria.
Wolf et al., 2017 [25]	U.S.A.	Prospective case-control study	417 patients (EoE = 120, healthy controls = 297)	Adults	BMI was lower in EoE cases than controls (25 kg/m^2^ vs. 28 kg/m^2^, *p* = 0.002), but it was not in the underweight range. Additionally, BMI was lower in EoE patients with esophageal narrowing, suggesting that a low weight in a patient suspected of having EoE should raise concern for esophageal remodeling.
Lee et al., 2015 [26]	U.S.A.	Cross-sectional study	57 EoE patients	Adults	The median BMI was 25.5 kg/m^2^, defined as overweight. There was no significant difference between the mean ages at diagnosis and different BMI categories (<25, 25–30, and >30 kg/m^2^). Rural and urban adult groups did not differ in BMI categories (24 kg/m^2^ ± 8.2 vs. 27 kg/m^2^ ± 11.7, *p* = 0.271).
Rezende et al., 2014 [27]	Brazil	Cross-sectional study	35 EoE patients	Children (<18 years)	A good nutritional state was observed in 82.8% of the enrolled children. In particular, 11.4% of enrolled children were overweight, whereas 5.7% were underweight.

BMI, body mass index; EoE, eosinophilic esophagitis; GERD, gastroesophageal reflux disease; PPI, proton pump inhibitor; WFL, weight-for-length.

**Table 3:** Studies reporting underweight and failure to thrive in children and adult patients with EGIDs.

Author, Year	Country	Study Design	Sample Size	Population	Outcomes
Hoofien et al., 2019 [39]	Europe	Multicentric retrospective study	410 EoE patients	Children	The most frequent indications for endoscopy were dysphagia (38%), gastroesophageal reflux (31.2%), food impaction (24.4%), and FTT (10.5%).
Chehade et al., 2018 [40]	U.S.A.	Multicentric study	705 EoE patients	Children and adults	FTT was present in 21.3% of enrolled subjects and was significantly common in children. Common pediatric comorbidities were neurological/developmental disorders, gastric tube placement, prematurity, atopic dermatitis, and food allergy.
Alhmoud et al., 2016 [41]	U.S.A.	Retrospective study	13 EoGE patients	Children and adults	FTT and weight loss were observed only in children. Two children (15%) had severe mucosal involvement leading to malabsorption, FTT, and weight loss.
Paquet et al., 2016 [42]	Canada	Retrospective study	62 EoE patients	Children	Sixty-two children were enrolled. Of these, 15 (24%) met at least one criterion for FTT.
Colson et al., 2014 [43]	France	Retrospective study	59 EoE patients	Children	Most children had negative WFH z scores, and 10% had nutritional indices compatible with moderate malnutrition. Nutrition therapy (elemental and six food elimination diets) did not impair nutritional status.
Spergel et al., 2009 [44]	U.S.A.	Retrospective study	620 EoE patients	Children	FTT/feeding issues and GERD-like symptoms were the most common presentations in the youngest children. (118 patients).

EoE, eosinophilic esophagitis; EoGE, eosinophilic gastroenteritis; FTT, failure to thrive; GERD, gastroesophageal reflux disease; WFL, weight-for-length.

**Table 4:** Potential factors that may negatively influence the nutritional status of patients with EGIDs.

Chronic esophageal inflammation leading to typical GI symptoms: recurrent vomiting and regurgitation, loss of appetite, food impaction, GERD-like symptoms
Diagnostic delay may increase the risk of esophageal stricture and prolong GI discomforting symptoms
The low compliance to therapies may sustain esophageal inflammation, also allowing a low grade of antigen exposure
Swallowing disorders and fear of food impaction may compromise feeding behavior, allowing the development of food avoidance, anorexia, and anxiety
Restrictive food-elimination diets may reduce adequate food oral intake and lead to low levels of vitamins
Atopic (IgE mediated food allergy, atopic dermatitis) and non-atopic comorbidities (CD, IBD, type 1 diabetes mellitus, ASDs, CF) may be associated with FTT, low growth, reduced food oral intake, vitamins deficiency, and undernutrition
Multisite GI eosinophilic inflammation with subsequent abnormal permeability may be a possible reason for nutrients loss and higher caloric and protein requirements in patients with EGIDs distal to the esophagus

ASDs, autism spectrum disorders; CD, coeliac disease; CF, cystic fibrosis; FTT, failure to thrive; GERD, gastroesophageal reflux disease; GI, gastrointestinal; IBD, inflammatory bowel disease.

**Table 5:** Studies reporting levels of vitamin D in children and adult patients with EoE.

Author, Year	Country	Study Design	Sample Size	Population	Outcomes
Mehta et al., 2018 [24].	U.S.A.	Prospective study	91 patients (GERD = 38, EoE = 53)	Children (0–7 years)	Enrolled children had adequate nutrient intakes, except for vitamin D levels that were low in both groups.
Slack et al., 2015 [54].	U.S.A.	Cross-sectional study	69 EoE patients	Children and adults	The median vitamin D level was 28.9 ng/mL. Patients with low vitamin D levels were older (25.5 years) and had a higher body mass index (25.2 kg/m^2^). Vitamin D insufficiency was not associated with IgE and surrogate markers of severity (dilation in adults or hospitalization or emergency visits in children).

EoE, eosinophilic esophagitis; GERD, gastroesophageal reflux disease.

**Table 6:** Useful questions to ask patients with EoE (Adapted from Muir et al., 2019) [59].

Does the patient take longer than others to eat?
Does the patient have to be reminded to chew a lot?
Does the patient need to cut food, especially steak, into small pieces?
Does the patient always need to drink during the meals?
Does the patient eat steak or crusty bread?

## References

[1] Licari A., Votto M., D’Auria E., Castagnoli R., Caimmi S.M.E., Marseglia G.L. (2020). Eosinophilic gastrointestinal diseases in children: A practical review. Curr. Pediatr. Rev..

[2] Cianferoni A., Spergel J.M. (2015). Eosinophilic esophagitis and gastroenteritis. Curr. Allergy Asthma Rep..

[3] Furuta G.T., Katzka D.A. (2015). Eosinophilic Esophagitis. N. Engl. J. Med..

[4] Dellon E.S., Liacouras C.A., Molina-Infante J., Furuta G.T., Spergel J.M., Zevit N., Spechler S.J., Attwood S.E., Straumann A., Aceves S.S. (2018). Updated International Consensus Diagnostic Criteria for Eosinophilic Esophagitis: Proceedings of the AGREE Conference. Gastroenterology.

[5] Spergel J.M., Dellon E.S., Liacouras C.A., Hirano I., Molina-Infante J., Bredenoord A.J., Furuta G.T. (2018). Participants of AGREE. Summary of the updated international consensus diagnostic criteria for eosinophilic esophagitis: AGREE conference. Ann. Allergy Asthma Immunol..

[6] O’Shea K.M., Aceves S.S., Dellon E.S., Gupta S.K., Spergel J.M., Furuta G.T., Rothenberg M.E. (2018). Pathophysiology of Eosinophilic Esophagitis. Gastroenterology.

[7] Votto M., Marseglia G.L., De Filippo M., Brambilla I., Caimmi S.M.E., Licari A. (2020). Early life risk factors in pediatric EoE: Could we prevent this modern disease?. Front. Pediatr..

[8] World Health Organization (WHO). https://www.who.int/news-room/q-a-detail/malnutrition.

[9] Tambucci R., Quitadamo P., Ambrosi M., De Angelis P., Angelino G., Stagi S., Verrotti A., Staiano A., Farello G. (2019). Association between obesity/overweight and functional gastrointestinal disorders in children. J. Pediatr. Gastroenterol. Nutr..

[10] Fifi A.C., Velasco-Benitez C., Saps M. (2020). Functional abdominal pain and nutritional status of children. A school-based study. Nutrients.

[11] Moher D., Liberati A., Tetzlaff J., Altman D.G., PRISMA Group (2009). Preferred reporting items for systematic reviews and meta-analyses: The PRISMA statement. BMJ.

[12] Lee E.Y., Yoon K.H. (2018). Epidemic obesity in children and adolescents: Risk factors and prevention. Front Med..

[13] Umano G.R., Pistone C., Tondina E., Moiraghi A., Lauretta D., Miraglia Del Giudice E., Brambilla I. (2019). Pediatric obesity and the immune system. Front. Pediatr..

[14] Figueroa-Munoz J.I., Chinn S., Rona R.J. (2001). Association between obesity and asthma in 4-11-year-old children in the UK. Thorax.

[15] Del Giudice M.M., Marseglia G.L., Leonardi S., Tosca M.A., Marseglia A., Perrone L., Ciprandi G. (2011). Fractional exhaled nitric oxide measurements in rhinitis and asthma in children. Int. J. Immunopathol. Pharmacol..

[16] Chen Y.C., Dong G.H., Lin K.C., Lee Y.L. (2013). Gender difference of childhood overweight and obesity in predicting the risk of incident asthma: A systematic review and meta-analysis. Obes. Rev..

[17] von Mutius E., Schwartz J., Neas L.M., Dockery D., Weiss S.T. (2001). Relation of body mass index to asthma and atopy in children: The National Health and Nutrition Examination Study III. Thorax.

[18] Boulet L.P. (2015). Obesity and atopy. Clin. Exp. Allergy.

[19] Zdanowicz K., Kucharska M., Sobaniec-Lotowska M.E., Lebensztejn D.M., Daniluk U. (2020). Eosinophilic Esophagitis in Children in North-Eastern Poland. J. Clin. Med..

[20] Alexander R., Alexander J.A., Akambase J., Harmsen W.S., Geno D., Tholen C., Katzka D.A., Ravi K. Proton pump inhibitor therapy in eosinophilic esophagitis: Predictors of nonresponse. Dig. Dis. Sci..

[21] Jensen E.T., Huang K.Z., Chen H.X., Landes L.E., McConnell K.A., Almond M.A., Safta A.M., Johnston D.T., Durban R., Jobe L. (2019). Longitudinal growth outcomes following first-line treatment for pediatric patients with eosinophilic esophagitis. J. Pediatr. Gastroenterol. Nutr..

[22] Kovačić M., Unić J., Mišak Z., Jadrešin O., Konjik V., Kolaček S., Hojsak I. (2019). One-year outcomes in children with eosinophilic esophagitis. Esophagus.

[23] Tanaka F., Fukumoto S., Morisaki T., Otani K., Hosomi S., Nagami Y., Kamata N., Taira K., Nakano A., Kimura T. (2019). Obesity and hiatal hernia may be non-allergic risk factors for esophageal eosinophilia in Japanese adults. Esophagus.

[24] Mehta P., Furuta G.T., Brennan T., Henry M.L., Maune N.C., Sundaram S.S., Menard-Katcher C., Atkins D., Takurukura F., Giffen S. (2018). Nutritional state and feeding behaviors of children with eosinophilic esophagitis and gastroesophageal reflux disease. J. Pediatr Gastroenterol. Nutr..

[25] Wolf W.A., Piazza N.A., Gebhart J.H., Rusin S., Covey S., Higgins L.L., Beitia R., Speck O., Woodward K., Cotton C.C. (2017). Association between body mass index and clinical and endoscopic features of eosinophilic esophagitis. Dig. Dis. Sci..

[26] Lee Y.J., Redd M., Bayman L., Frederickson N., Valestin J., Schey R. (2015). Comparison of clinical features in patients with eosinophilic esophagitis living in an urban and rural environment. Dis. Esophagus.

[27] Rezende E.R., Barros C.P., Ynoue L.H., Santos A.T., Pinto R.M., Segundo G.R. (2014). Clinical characteristics and sensitivity to food and inhalants among children with eosinophilic esophagitis. BMC Res. Notes.

[28] Licari A., Votto M., Scudeller L., De Silvestri A., Rebuffi C., Cianferoni A., Marseglia G.L. (2020). Epidemiology of nonesophageal eosinophilic gastrointestinal diseases in symptomatic patients: A systematic review and meta-analysis. J. Allergy Clin. Immunol. Pract..

[29] Silva F.M.C.E., Oliveira E.E., Ambrósio M.G.E., Ayupe M.C., Souza V.P., Gameiro J., Reis D.R.L., Machado M.A., Macedo G.C., Mattes J. (2020). High-fat diet-induced obesity worsens TH2 immune response and immunopathologic characteristics in murine model of eosinophilic oesophagitis. Clin. Exp. Allergy.

[30] Pensabene L., Salvatore S., D’Auria E., Parisi F., Concolino D., Borrelli O., Thapar N., Staiano A., Vandenplas Y., Saps M. (2019). Cow’s milk protein allergy in infancy: A risk factor for functional gastrointestinal disorders in children?. Nutrients.

[31] Cheng E., Souza R.F., Spechler S.J. (2014). Eosinophilic esophagitis: Interactions with gastroesophageal reflux disease. Gastroenterol. Clin. N. Am..

[32] Tobey N.A., Hosseini S.S., Argote C.M., Dobrucali A.M., Awayda M.S., Orlando R.C. (2004). Dilated intercellular spaces and shunt permeability in nonerosive acid-damaged esophageal epithelium. Am. J. Gastroenterol..

[33] Cao W., Cheng L., Behar J., Fiocchi C., Biancani P., Harnett K.M. (2004). Proinflammatory cytokines alter/reduce esophageal circular muscle contraction in experimental cat esophagitis. Am. J. Physiol. Gastrointest. Liver Physiol..

[34] El-Serag H.B., Graham D.Y., Satia J.A., Rabeneck L. (2005). Obesity is an independent risk factor for GERD symptoms and erosive esophagitis. Am. J. Gastroenterol..

[35] Vaishnav B., Bamanikar A., Maske P., Reddy A., Dasgupta S., Dasgupta S. (2017). Gastroesophageal reflux disease and its association with body mass index: Clinical and endoscopic study. J. Clin. Diagn. Res..

[36] Nam S.Y., Choi I.J., Ryu K.H., Park B.J., Kim Y.-W., Kim H.B., Kim J. (2015). The effect of abdominal visceral fat, circulating inflammatory cytokines, and leptin levels on reflux esophagitis. J. Neurogastroenterol. Motil..

[37] Lim H.-S., Kim S., Hong S.J. (2018). Food Elimination Diet and Nutritional Deficiency in Patients with Inflammatory Bowel Disease. Clin. Nutr. Res..

[38] Liacouras C.A., Spergel J., Gober L.M. (2014). Eosinophilic esophagitis: Clinical presentation in children. Gastroenterol. Clin. N. Am..

[39] Hoofien A., Dias J.A., Malamisura M., Rea F., Chong S., Oudshoorn J., Nijenhuis-Hendriks D., Otte S., Papadopoulou A., Romano C. (2019). Pediatric Eosinophilic Esophagitis: Results of the European Retrospective Pediatric Eosinophilic Esophagitis Registry (RetroPEER). J. Pediatr. Gastroenterol. Nutr..

[40] Chehade M., Jones S.M., Pesek R.D., Burks A.W., Vickery B.P., Wood R.A., Leung D.Y., Furuta G.T., Fleischer D.M., Henning A.K. (2018). Phenotypic Characterization of Eosinophilic Esophagitis in a Large Multicenter Patient Population from the Consortium for Food Allergy Research. J. Allergy Clin. Immunol. Pract..

[41] Alhmoud T., Hanson J.A., Parasher G. (2016). Eosinophilic Gastroenteritis: An Underdiagnosed Condition. Dig. Dis. Sci..

[42] Paquet B., Bégin P., Paradis L., Drouin E., Roches A.D. (2016). High rate of failure to thrive in a pediatric cohort with eosinophilic esophagitis. Ann. Allergy Asthma Immunol..

[43] Colson D., Kalach N., Soulaines P., Vannerom Y., Campeotto F., Talbotec C., Chatenoud L., Hankard R., Dupont C. (2014). The impact of dietary therapy on clinical and biologic parameters of pediatric patients with eosinophilic esophagitis. J. Allergy Clin. Immunol. Pract..

[44] Spergel J.M., Brown-Whitehorn T.F., Beausoleil J.L., Franciosi J., Shuker M., Verma R., A Liacouras C. (2009). 14 years of eosinophilic esophagitis: Clinical features and prognosis. J. Pediatr. Gastroenterol. Nutr..

[45] Schoepfer A.M., Safroneeva E., Bussmann C., Kuchen T., Portmann S., Simon H.U., Straumann A. (2013). Delay in diagnosis of eosinophilic esophagitis increases risk for stricture formation in a time-dependent manner. Gastroenterology.

[46] Votto M., Castagnoli R., De Filippo M., Brambilla I., Cuppari C., Marseglia G.L., Licari A. (2020). Behavioral issues and quality of life in children with eosinophilic esophagitis. Minerva Pediatr..

[47] Mehta H., Groetch M., Wang J. (2013). Growth and nutritional concerns in children with food allergy. Curr. Opin. Allergy Clin. Immunol..

[48] Wu Y.P., Franciosi J.P., Rothenberg M.E., Hommel K.A. (2012). Behavioral feeding problems and parenting stress in eosinophilic gastrointestinal disorders in children. Pediatr. Allergy Immunol..

[49] Mukkada V.A., Haas A., Maune N.C., Capocelli K.E., Henry M., Gilman N., Petersburg S., Moore W., Lovell M.A., Fleischer D.M. (2010). Feeding dysfunction in children with eosinophilic gastrointestinal diseases. Pediatrics.

[50] Pesek R.D., Reed C.C., Muir A.B., Fulkerson P.C., Menard-Katcher C., Falk G.W., Kuhl J., Martin E.K., Magier A.Z., Consortium of Eosinophilic Gastrointestinal Disease Researchers (CEGIR) (2019). Increasing rates of diagnosis, substantial co-occurrence, and variable treatment patterns of eosinophilic gastritis, gastroenteritis, and colitis based on 10-year data across a multicenter consortium. Am. J. Gastroenterol..

[51] Capucilli P., Hill D.A. (2019). Allergic Comorbidity in Eosinophilic Esophagitis: Mechanistic Relevance and Clinical Implications. Clin. Rev. Allergy Immunol..

[52] Capucilli P., Cianferoni A., Grundmeier R.W., Spergel J.M. (2018). Comparison of comorbid diagnoses in children with and without eosinophilic esophagitis in a large population. Ann. Allergy Asthma Immunol..

[53] Mailhot G., White J.H. (2020). Vitamin D and Immunity in Infants and Children. Nutrients.

[54] Slack M.A., Ogbogu P.U., Phillips G., Platts-Mills T.A., Erwin E.A. (2015). Serum vitamin D levels in a cohort of adult and pediatric patients with eosinophilic esophagitis. Ann. Allergy Asthma Immunol..

[55] Linglart A., Rothenbuhler A., Adamsbaum C., Colson D., Soulaines P., Dupont C. (2015). The impact of pediatric eosinophilic esophagitis on bone metabolism. J. Allergy Clin. Immun..

[56] Waterhouse K., Katta A., Teckman J., Foy T., Derdoy J., Jain A., Knutsen A., Becker B. (2011). Vitamin D levels in children with eosinophilic esophagitis and gastroesophageal reflux. J. Allergy Clin. Immun..

[57] Fissinger A., Mages K.C., Solomon A.B. (2020). Vitamin deficiencies in pediatric eosinophilic esophagitis: A systematic review. Pediatr. Allergy Immunol..

[58] Noimark L., Cox H.E. (2008). Nutritional problems related to food allergy in childhood. Pediatr. Allergy Immunol..

[59] Muir A.B., Brown-Whitehorn T., Godwin B., Cianferoni A. (2019). Eosinophilic esophagitis: Early diagnosis is the key. Clin. Exp. Gastroenterol..

[60] Slack I.F., Schwartz J.T., Mukkada V.A., Hottinger S., Abonia J.P. (2020). Eosinophilic Esophagitis: Existing and Upcoming Therapies in an Age of Emerging Molecular and Personalized Medicine. Curr. Allergy Asthma Rep..

[61] Venkatesh R.D., Dellon E.S. (2020). This String’s Attached: The Esophageal String Test for Detecting Disease Activity in Eosinophilic Esophagitis. Gastroenterology.

[62] Walker M.M., Potter M., Talley N.J. (2018). Eosinophilic gastroenteritis and other eosinophilic gut diseases distal to the oesophagus. Lancet Gastroenterol. Hepatol..

[63] Cotton C.C., Eluri S., Wolf W.A., Dellon E.S. (2017). Six-food elimination diet and topical steroids are effective for eosinophilic esophagitis: A meta-regression. Dig. Dis. Sci..

[64] Licari A., Castagnoli R., Marseglia A., Olivero F., Votto M., Ciprandi G., Marseglia G.L. (2020). Dupilumab to Treat Type 2 Inflammatory Diseases in Children and Adolescents. Paediatr. Drugs.

